# Comprehensive analysis of early pregnancy loss based on cytogenetic findings from a tertiary referral center

**DOI:** 10.1186/s13039-021-00577-8

**Published:** 2021-12-04

**Authors:** Xiaoqing Wu, Linjuan Su, Xiaorui Xie, Deqin He, Xuemei Chen, Meiying Wang, Linshuo Wang, Lin Zheng, Liangpu Xu

**Affiliations:** 1 Medical Genetic Diagnosis and Therapy Center of Fujian Provincial Maternity and Child Health Hospital, Affiliated Hospital of Fujian Medical University, Fujian Provincial Key Laboratory for Prenatal Diagnosis and Birth Defect, No. 18 Daoshan Road, Fuzhou, 350001 Fujian China; 2 Department of Laboratory Medicine, Fujian Medical University, No. 88 Jiaotong Road, Fuzhou, 350002 Fujian China

**Keywords:** Products of conception, Chromosomal abnormalities, Maternal age, Live birth history, Previous miscarriage, Mode of conception

## Abstract

**Background:**

Pregnancy loss is one of the most common complications during pregnancy. Clinical consultation based on etiology analysis are critical for reducing anxiety and distress. This study aimed to perform a comprehensive analysis for products of conception (POC) in miscarriage based on genetic etiology and clinical information.

**Methods:**

A retrospective study was conducted according to cytogenetic findings of 1252 POC from spontaneous pregnancy loss over 11 years. The frequencies and profiles of chromosomal abnormalities were discussed according to the classification of women with different maternal ages, previous miscarriage history, normal live birth history, and different modes of conception.

**Results:**

A total of 667 (53.2%) chromosomal abnormalities were observed, including 592 (47.3%) cases of numerical abnormalities, 38 (3.0%) cases of structural abnormalities, and 37 (3.0%) cases of mosaic aberrations. In women above 40 years of age, the rates of chromosomal abnormalities and viable autosomal trisomy were significantly higher than those in women with ≤ 29, 30–34, and 35–39 years of age (*p* < 0.05). The frequency of abnormal karyotype in women with normal live birth history was 61.1%, significantly higher than 52.5% in women without normal live birth history (*p* < 0.05). There was no significant differences among women without, with 1–2, and ≥ 3 previous miscarriages regarding the rate of abnormal karyotype (*p* > 0.05); viable autosomal trisomy was less common in women with ≥ 3 previous miscarriages than women with < 3 miscarriages. The frequency of chromosomal abnormalities was 49.0% and 55.0% in women with assisted conception and natural conception (*p* > 0.05), respectively; monosomy X was more frequently detected in women with natural conception than assisted conception.

**Conclusion:**

The frequencies and profiles of chromosomal abnormalities in early miscarriages are strongly associated with clinical information including maternal age, previous miscarriage, live birth history, and mode of conception. Cytogenetic analysis of POC should be recommended to women with a first miscarriage and women with normal live birth history.

## Background

Pregnancy loss before 12 weeks of gestation is the most common complication of pregnancy and has a substantial impact on a couple’s physical and psychological well-being. The frequency was estimated to be 10–15% of all pregnancies [1]. Understanding the reason for miscarriage is of great benefit to support clinical consultants as well as medical management for future reproductive planning. Abnormal embryonic karyotypes are recognized as the most important and detectable factors [2–5]. De novo numerical abnormalities, especially autosomal trisomies, may explain a vast proportion of recurrent spontaneous miscarriage [6]. Therefore, in our center, G-banding karyotyping is sometimes more acceptable owing to its detectability of numerical anomalies and lower cost than molecular methodology. Most genetic anomalies that lead to miscarriage are sporadic, probably induced by random errors during gametogenesis, and they seem to have no association with future pregnancies. However, some numerical abnormalities may indicate a potential recurrence risk of chromosomal abnormality, especially for viable autosomal trisomies including trisomy 13 (T13), trisomy 18 (T18), and trisomy 21 (T21). Viable autosomal trisomies are the major target chromosomal abnormalities in prenatal screening and diagnosis, while their occurrence in early pregnancy loss was rarely discussed.

There was sufficient evidence for the necessity of genetic analysis of POC in patients with recurrent miscarriage [7–9]. However, in our clinical practice, for women who were experiencing the first pregnancy loss, or who already had a normal child, the chromosomal analysis of POC was not routinely conducted. In this study, we comprehensively investigated the associations between chromosomal abnormalities of POC and clinical information containing maternal age, history of miscarriage, normal live birth history, as well as the mode of conceptions, to evaluate the influence of these factors on the frequencies and profiles of genetic abnormalities in early pregnancy loss.

## Results

**Fig. 1:**
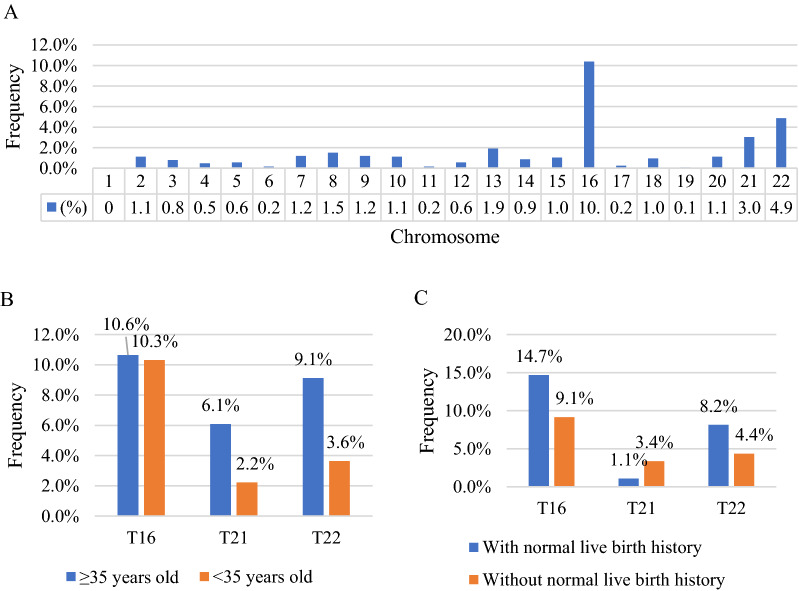
Distribution profile and frequency of autosomal trisomy involved in miscarriage samples. **A** Distribution profile and frequency of autosomal trisomy involved in the total cohort. All chromosomes except chromosome 1 were involved in trisomies, with trisomy 16 being the most common finding, followed by trisomy 22 and trisomy 21. **B** Comparison in the frequencies of T16, T21and T22 miscarriage between women aged ≥ 35 years old and < 35 years old. **C** Comparison in the frequencies of T16, T21and T22 miscarriage between women with and without normal live birth history

None of the 100 samples that underwent QF-PCR showed maternal cell contamination. Abnormal karyotypes were detected in 667 out of 1252 (53.2%) cases, including 592 (47.3%) numerical abnormalities, 38 (3.0%) cases of structural abnormalities, and 37(3.0%) cases of mosaic abnormalities (Table 1). The rates of viable autosomal trisomy including T21, T13, and T18 were 3.0%, 1.9%, and 1.0%, respectively. Other autosomal trisomies occurred in 27.3% of the total cases. All chromosomes except chromosome 1 were involved in trisomies, with T16 being the most common finding, followed by T22, and trisomy T21 (Fig. 1A). Monosomy X was the most frequently encountered sex chromosomal abnormality and the incidence was 6.8%. Among 38 cases of structural abnormalities, 33 were unbalanced rearrangements with 10 of them being derivated from parental balanced rearrangement, and 22 of them being incidental; 5 cases were parentally inherited balanced rearrangement (Table 2).

**Table 1:** The details of 667 cases of chromosomal abnormalities

	Numbers (n)	Frequency in 667 cases with chromosomal abnormalities (%)	Frequency in total cases (%)
*Numerical abnormalities*			
Trisomy 21	38	5.5	3.0
Trisomy 13	24	3.6	1.9
Trisomy 18	12	1.8	1.0
Other autosomal trisomy	343	51.2	27.3
Other autosomal aneuploidy	20	3.0	1.6
45,X	75	11.2	6.8
47,XXY	4	0.6	0.3
Triploidy	47	7.0	3.8
Hypotriploidy or hypertriploidy	8	1.2	0.6
Tetraploidy	19	2.8	1.5
Hypotetraploidy or hypertetraploidy	2	0.3	0.2
Total	592	88.8	47.3
*Structural abnormalities*			
Unbalanced structural abnormalities	33	4.9	2.6
Balanced rearrangement	5	0.7	0.4
Total	38	5.7	3.0
*Mosaic abnormalities*	37	5.5	3.0

**Table 2:** Details of 38 cases with structural abnormalities

Case	Maternal age (years)	Previous miscarriage	Karyotype
1	26	NV	46,XX,der(14)t(2;14)(p23;q32)dpat
2	31	NV	46,XY,der(5)t(5;?5)(p14;q14) dn
3	28	NV	47,XY,+der(17)t(17;19)(q21;q13.4)dmat
4	35	NV	46,XX,del(8)(p12)dn
5	37	NV	46,Y,i(Xq)dn
6	27	NV	46,X,add(X)(q?23)dn
7	31	NV	46,XY,add(5)(p15) dn
8	26	1	46,X,der(X)t(X;8)(p21;q11),-8 dn
9	31	1	46,XY,add(8)(p22) dn
10	27	2	46,X,del(X)(q22) dn
11	33	2	46,XY,psu idic(15)(q26.3) dn
12	28	5	46,XY,der(1)t(1;13)(p36;q12)dpat
13	31	2	46,XX,del(8)(p11.2)dn
14	30	1	46,XX,del(8)(p?12)^a^
15	19	2	46,XY,der(10)t(10;21)(p14;q21)dmat
16	26	1	46,XY,der(8)t(4;8)(q27;p12) dpat
17	27	2	46,XX,add(5)(p14)dn
18	26	1	46,XX,add(10)(q25) dn
19	29	0	46,XX,add(8)(p22) dn
20	31	1	47,XY,+der(2)t(2;11)(p23;q13.1) dn
21	24	0	46,XY,der(8)t(8;?20)(p11.2;p12) dn
22	30	1	46,XX,del(11)(p13) dn
23	28	0	46,XX,add(7)(q36)dn
24	28	1	46,del(5)(p14)dn
25	31	0	46,XX,del(8)(p12)dn
26	26	1	46,XY,der(15)t(6;15)(q13;p11.2)dmat
27	31	0	46,XX,der(12)t(11;12)(q24;q23)dmat
28	27	2	46,XX,rec(4)dup(4q)inv(4)(p16.1q12)dmat
29	25	0	46,XX,del(8)(?p12) dn
30	28	1	46,XY,add(5)(p15) dn
31	25	1	46,XY,der(4)t(4;6)(p15;q23)dmat
32	31	2	46,XY,der(l5)t(1;15)(p36.3;q26.1)dmat
33	27	0	46,XY,del(8)(p21) dn
34	23	NV	46,XX,ins(11;13)(q23;q22q32) mat
35	28	3	45,XY,rob(13;14) (q10;q10)mat
36	31	NV	45,XX,rob(14;15)(q10;q10)pat
37	32	2	46,XX,t(1;15)(q44;q14) mat
38	38	1	45,XY,rob(13;14)(q10;q10)mat

NV, not available

^a^Parental karyotype is unknown

The associations between abnormal karyotype and maternal age, previous miscarriages, live birth history, and mode of conception are presented in Table 3. Similar incidences of chromosomal abnormalities were found among women aged ≤ 29, 30–34, and 35–39 years (*p* > 0.05), and they were all significantly lower than that in women ≥ 40 years old (*p* < 0.05). The frequency of viable autosomal trisomy increased with the maternal age, while the incidence of monosomy X decreased with the maternal age. Of the three most frequently encountered trisomies described above, the incidences of T21 and T22 in women with advanced maternal age (≥ 35 years old) were significant higher than those in others (*p* < 0.05) (Fig. 1B). Similar frequencies of abnormal karyotype were observed in women without, with 1-2 and ≥3 previous miscarriages (*p* > 0.05). In women with ≥3 previous miscarriage, the rate of viable autosomal trisomy was significantly lower than that in women with <3 previous miscarriage (*p* < 0.05). The chromosome abnormal rate in women with normal live birth history were higher than that in women without normal live birth history (p < 0.05); the rates of T16 and T22 were higher in women with normal live birth history than those in women without normal live birth history (*p* < 0.05) (Fig. 1C). Concerning different modes of conception, women with assisted pregnancy had a lower incidence of monosomy X than women with natural pregnancy (*p* < 0.05).

**Table 3:** Association between clinical information and the frequency of chromosomal abnormalities

	Abnormal karyotype (n, %)	Viable autosomal trisomy (n, %)	Monosomy X (n, %)
*Maternal age (years) (N* = *1252)*			
≤ 29 (n = 537)	279, 52.0%,	24, 4.5%	34, 6.3%
30–34 (n = 452)	230, 50.9%	24, 5.3%	27, 6.0%
35–39 (n = 201)	115, 57.2%,	14, 7.0%	11, 5.5%
≥ 40 (n = 62)	43, 69.4%,	12, 19.4%	3, 4.8%
*p*	< 0.05	< 0.05	> 0.05
*Normal live birth history (N* = *895)*			
Yes (n = 185)	113, 61.1%	12, 6.5%	11, 5.9%
No (n = 710)	373, 52.5%	50, 7.0%	41, 5.8%
*p*	< 0.05	> 0.05	> 0.05
*Previous miscarriage (N* = *895)*			
0 (n = 273)	146, 53.5%	20, 7.3%	11, 4.0%
1–2 (n = 505)	271, 53.8%	40, 7.9%	33, 6.5%
≥ 3 (n = 117)	69, 59.0%	2, 1.7%	8, 6.8%
*p*	> 0.05	< 0.05	> 0.05
*Mode of conception (N* = *895)*			
Assisted conception (n = 115)	57, 49.0%	8, 7.0%	1, 0.9%
Natural conception (n = 780)	429, 55.0%	54, 6.9%	51,6.5%
*P*	> 0.05	> 0.05	< 0.05

## Discussion

Although various molecular methodology have been applied in the genetic analysis of POC, we focused on the cytogenetic results due to the following reasons: first, in clinical practice, conventional karyotyping was still opted by many women due to lower cost compared to molecular testing; second, large fragmental abnormalities are lethal, and contribute to miscarriage [10], while submicroscopic aberrations could be viable and contribute to prenatal ultrasound anomalies or neurodevelopmental disorders [11].

Consistent with previous reports, nearly half of the pregnancy loss were attributed to fetal chromosomal anomalies, with the vast majority of them being numerical anomalies [12]. Autosomal trisomies composed a significantly large proportion. In line with previous reports [1, 4, 13, 14], chromosomes 1 and 19 were rarely involved in trisomies, which may indicate that, chromosome 1 and 19 require more precise dosage for early embryonic development. Apart from numerical anomalies, structural abnormalities accounted for approximately 3.0% of the total abnormalities. Study of parental origin is essential for cases with balanced or unbalanced rearrangements. In our study, 15 out of 33 POC with structural abnormalities were confirmed to be parental origin. Their numbers of miscarriage ranged from 0 to 5. Approximately 2.7~6.7% of couples who have experienced recurrent spontaneous abortions are balanced chromosomal aberration carriers [15]. Our results suggests that when chromosome structural abnormality is found in POC, parental karyotyping is strongly recommended, no matter whether it is the first pregnancy loss, and whether there is a balanced or unbalanced structural abnormality.

Advanced maternal age ( ≥ 35 ) is a well-known independent factor associated with the frequencies of cytogenetic abnormalities in early miscarriages [1, 13, 16]. In this study, the frequencies of abnormal karyotype in women aged up to 30 years, 30–34 years, as well as 35–39 years similar, but all of them were significantly lower than that in women ≥ 40 years old. The tendency was in line with that of viable autosomal trisomy, which confirmed the close association between maternal age and viable autosomal trisomy. In Hassold’s report [17], the age effect was not pronounced for all trisomic miscarriage. In our study, T16, T22 and T21 were the top three frequent trisomies; T21 and T22 were more commonly observed in women with advanced maternal age (≥ 35 years old) than young maternal age (< 35 years old), while the occurrence of T16 in POC was not affected by advanced age. This observation supported the view that T16 had special age-independent factors associated with its frequency in spontaneous abortion [17]. Monosomy X was the most commonly encountered viable sex chromosomal abnormality. Unlike viable autosomal trisomy, the frequency of monosomy X was not increased with the maternal age, in agreement with previous reports [1, 18, 19]. Hassold et al. [20, 21] found that paternal sex chromosome loss was the most common error leading to 45, X. They speculated that monosomy X was more likely to be derived from meiotic error of the father rather than the mother. The inverse age effect for monosomy X has also been observed in an earlier study by Warburton et al. [22]. Two possible reasons have been raised: increase in the frequency of monosomy X conceptions related to events in meiosis, fertilization, or early zygotic division, or increase in the rate of survival of monosomy X conceptions to the stage of being recognizable pregnancies.

To the best of our knowledge, the association between the normal live birth history and chromosomal abnormalities in POC was rarely discussed. In our clinical practice, patients who have given birth to normal children always thought that they were unlikely to be pregnant with chromosomal abnormalities in the next pregnancy. However, the rate of abnormal karyotype in women with normal live birth history was significantly higher than that in women without it. Women with normal live birth history had significantly higher rates of miscarriage involving T16 and T22, and similar incidence of miscarriage involving T21, compared to women without normal live birth history. Maternal age factor, but not all, may play an important role in the result. Because women with a history of normal live birth tend to be older, especially under the two-child policy in China, which resulted in an increasing proportion of women with advanced maternal age [23, 24]. Therefore, genetic testing is also valuable for women with a history of normal live births. In addition, the relationship between the number of previous miscarriages and chromosomal abnormality rate was controversial. Some researchers concluded that women with first miscarriage would not raise the rate of chromosome anomaly in the next pregnancy, and suggested that detailed investigation of the reasons might not be necessary for the first miscarriage [5]. In certain studies, there was a lower frequency of chromosomal abnormalities in recurrent miscarriages than that in first miscarriages [19, 25–27], whereas in some reports, the frequency of chromosomal abnormalities did not change with the number of miscarriage [28–30]. In our study, the cytogenetic abnormality rates in the first miscarriage and recurrent miscarriage with two or more miscarriages all exceeded 50%, and they showed no significant differences. We think that cytogenetic tests should be offered to the couples experiencing the first miscarriage. For the viable autosomal trisomy, the frequency was remarkably decreased in women with ≥3 miscarriage. We concluded that viable autosomal trisomy less likely occurred in pregnancy loss after 3 miscarriage history.

Assisted reproductive technology (ART) enables infertile couples to achieve pregnancy. However, miscarriage is still inevitable. The prevalence of early miscarriage following ART ranges from 22–63% and one of the major causes is embryonic chromosomal abnormality [31, 32]. Whether assisted conception increases or decreases the risk of chromosomal abnormalities in early spontaneous abortion is controversial. Our study showed no significant differences in the frequencies of abnormal karyotypes or viable autosomal trisomy between assisted conception and natural conception. But the rate of monosomy X was significantly lower in the assisted conception group. It may be explained by different ART treatments. Previous studies demonstrated that the incidence of monosomy X was significantly higher in abortus following intracytoplasmic sperm injection (ICSI) treatment, compared to that following in vitro fertilization (IVF) [33, 34]. The mechanism is unclear. It is possible that the damage to the cytoskeleton caused by injection leads to mitotic errors [35] or that the preferential location of the X chromosome in the subacrosomal region of the sperm nucleus is related to reduced DNA decondensation and its propensity for inactivation [36]. Therefore, we believe that the reason for the low frequency of detection of monosomy X could be attributed to the low proportion of ICSI patients in the assisted reproduction group; but it could not be confirmed owing to the lack of information on the ART used in our study.

Some limitations might lead to biased results of the study. First, medical information was not available for all cases. Second, maternal cell contamination was not evaluated for all samples.

## Conclusion

The frequencies and profiles of chromosomal abnormalities in early miscarriages samples are strongly associated with clinical factors including maternal age, previous miscarriage, live birth history, and mode of conception. Chromosomal analysis of POC should be recommended even in women with first miscarriage, and women with normal live birth history.

## Materials and methods

### Data resources

This is a retrospective study of chromosomal analysis from 1430 patients with early pregnancy loss who underwent curettage procedures between April 2009 and September 2020. Excluding 178 cases of cultural failure, a total of 1252 POC including 1250 chorionic villi and 2 fetal tissues were enrolled. The mean age of the patients was 35.5 years old, ranging from 19 to 47 years, and the mean gestational age was 10.1 weeks, ranging from 7 to 14 weeks.

Clinical information including early miscarriage history, normal live birth history, and mode of conception was not correctly recorded in 357 cases, thus it was available in only 895 cases. Maternal age was classified into the following four groups: ≤ 29, 30–34, 35–39, and ≥ 40 years of age. The numbers of previous early miscarriages were classified into three groups: 0, 1–2, and ≥ 3. The normal live birth history was categorized as “0” and “≥ 1” groups. The mode of conception was categorized as groups of assisted conception and natural conception.

The present study was approved by the Protection of Human Ethics Committee of Fujian Provincial Maternity and Children’s Hospital, affiliated Hospital of Fujian Medical University. Written informed consent was obtained from individual or guardian participants.

### Conventional karyotyping and maternal cell contamination evaluation

The specimens were carefully rinsed with sterile physiological saline and dissected from blood, clot, and maternal decidua base on operation experience. Cell culture and G-banded karyotyping were performed according to the standard protocols in our laboratory. The specimens were cultivated for about 9–14 days, then arrested in metaphase, and finally, Wright's stain was used for G-banding at a resolution of 320-400 banding. Karyograms were prepared using CytoVision, a computer-assisted karyotyping system (Leica Biosystems, Newcastle, UK). Parental karyotyping was offered to all couples whose POC revealed structural chromosome abnormalities. Before 2019, maternal cell contamination evaluation was conducted by selecting typical villi through morphological identification to prevent contamination of decidual tissue. Thus maternal peripheral blood was obtained in only 100 cases for the quantitative fluorescent-polymerase chain reaction (QF-PCR) to exclude maternal cell contamination after 2019.

### QF-PCR

DNA from maternal blood and POC was extracted using a QIAGEN kit (Qiagen, Hilden, Germany) according to the manufacturer's instructions. Multiple QF-PCR was performed using Chromosome (13/18/21/X/Y) multiplex STR Genotyping Kit (Guangzhou Darui Biotechnology Co., Ltd.) containing 20 STR markers (fourteen STR markers on autosomes 13, 18, and 21, four on the chromosome X‐linked markers, one on amelogenin, and SRY on chromosome Y). PCR products were separated on an ABI 3500 (Applied Biosystems, Foster City, CA, USA) capillary genetic analyser, and results were analyzed by ABI genemapper 6.0. The informative markers presented in the POC DNA sample were compared to those in a maternal DNA sample to estimate the presence of maternal cell contamination.

### Statistical analysis

All data were entered into a Microsoft Excel 2016 (Microsoft Corp., Redmond, WA) spreadsheet, and SPSS software version 26.0 (SPSS, Inc., Chicago, IL) was used for statistical analysis. Statistical comparisons were performed using chi-square test, and *p* < 0.05 was considered statistically significant.

## Data Availability

The datasets used and/or analyzed during the current study are available from the corresponding author on reasonable request.
